# Notes and News

**Published:** 1896-07-04

**Authors:** 


### Notes and News.

(Collected and Communicated.)

Subscriptions are needed towards the completion of the statue of the late Surgeon-Major Parke. They may be sent to the British Medical Association.

There seems to be a serious intention of re-organising the accommodation of the out-patients' department at St. Thomas's Hospital, the present space being found very inadequate.

Mr. Walker, who has lately resigned the stewardship of St. Thomas's Hospital, has been presented with a silver salver in recognition of his valuable services to the institution.

Surgeon-Major Heuston, of the British Army Medical Staff, who was appointed first professor in charge of the Chinese Imperial Medical College at Tientsin, Pekin, about two and a-half years ago, has been decorated by the Emperor of China with the Order of the Double Dragon, which carries the rank of mandarin.

A new hospital has been opened at Naini Tal, Allahabad, in India. The main building consists of two distinct hospitals, one on the ground floor for males, and one on the upper floor for females. Each has a separate entrance, dispensary, consulting and operating rooms, and accommodation for fifteen in-patients. The new hospital is called the Crossethwaite Hospital.

The relations between the University of Cambridge and Addenbrooke's Hospital are now placed on a much more favourable basis than has hitherto existed. Under the scheme recently adopted by the governors of the hospital lectures may be delivered in the museum or surgery of the hospital, which may be illustrated by the cases under treatment in the institution. Professors Allbutt and Sir G. Humphrey will be the first to take advantage of the new rule.

It is announced that Princess Louise will open a bazaar at Crewe Hall on August 6th in aid of the Crewe Memorial Cottage Hospital Endowment Fund.

H.R.H. the Duke of York has consented to be a patron of the Royal Hospital, Richmond, and has expressed his intention of being present on July 8th, on the occasion of the opening of the "Princess May's Ward for Children" by H.R.H. the Duchess of York.

At the annual meeting of the Medical Missionary Association, it was stated that 215 medical missionaries are now at work in different parts of the world, by whom much good work is done. Dr. George Saunders, C.B., has been elected president, in place of the late Dr. Lockhart, the first medical missionary to China.

The Metropolitan Hospital Sunday Fund collection for the present year now reaches £37,200. Amongst the contributing congregations are those of St. Peter's, Eaton Square, £981; All Saints', Ennismore Gardens, £507; St. Paul's, Knightsbridge, £319; and St. Martin's-in-the-Fields, £200.

Only thirteen fresh cases of small-pox were notified in Gloucester last week, and the city now claims to contain the best-vaccinated community in England. The new water supply, too, is now completed, and proves most satisfactory, so that, the last remaining effects of the epidemic over, Gloucester may look forward to a most favourable sanitary condition.

H.R.H. the Duchess of Albany will honour the London Orphan Asylum, Watford, with a visit on July 18th for the purpose of distributing the prizes to the children of that institution. This society does an admirable work, and well deserves the support of the benevolent. It receives destitute orphans, maintains, clothes, and educates them, and eventually places them in situations. This work costs about £28,000 a-year, of which upwards of £15,000 has to be obtained from voluntary sources. There are at present 500 orphan children in the institution.

The total receipts from the concert at the Queen's Hall on June 11th, in aid of the Ladies' Association Endowment Fund, Great Northern Central Hospital, were just over £1,100. Nothing will be deducted for expenses. The purses presented to the Duchess of York amounted to £1,843, including the generous gift of £1,050 sent anonymously through Mrs. Cory-Wright. The total net receipts from all sources are £2,943, and as a result two beds have been endowed, and a considerable surplus remains in hand, for the further benefit of the Ladies' Endowment Fund, at Messrs. Coutts's bank.

The Prince and Princess of Wales, accompanied by the Princesses Maud and Victoria and Prince Charles of Denmark, attended the 30th anniversary of Dr. Barnardo's Homes at the Royal Albert Hall last week. The hall was filled in every part. The programme, illustrative of the arts and crafts pursued under Dr. Barnardo's direction, was of a varied character. Purses of £5 and upwards were received by the Princess of Wales, and a donation of £200 was announced from Lord Brassey. The Duchess of Sutherland presented prizes to former inmates of the Homes who had kept situations with credit for periods varying from one to ten years.

